# Unveiling the potential of *Aspergillus terreus* SJP02 for zinc remediation and its driving mechanism

**DOI:** 10.1038/s41598-025-87749-3

**Published:** 2025-01-27

**Authors:** Shobham, Vishalakshi Bhanot, Mamta, Sanjay Kumar Verma, Suresh Gupta, Jitendra Panwar

**Affiliations:** 1 Department of Biological Sciences, Birla Institute of Technology and Sciences, Pilani, 333031 Rajasthan India; 2 Department of Chemical Engineering, Birla Institute of Technology and Science, Pilani, 333031 Rajasthan India

**Keywords:** Wastewater, Zinc, Mycoremediation, Sorption, Process parameters, *Aspergillus Terreus* SJP02, Microbiology, Environmental sciences

## Abstract

In present study, 15 morphologically different fungi isolated from rhizopheric soils of an industrial area were screened for their Zn^2+^ removal efficiency from aqueous solution. Isolate depicting highest potential was molecularly identified as *Aspergillus terreus* SJP02. Effect of various process parameters viz. biosorbent dose, contact time, temperature, agitation rate, pH and initial Zn^2+^ concentration on the fungal sorption capacity were studied. The biosorbent exhibited maximum Zn^2+^ sorption capacity of 10.7 *±* 0.2 mg g^− 1^ in 60 min. Desorption studies showed 71.46% Zn^2+^ recovery rate in 120 min with 0.01 N HNO_3_, indicating efficient metal recovery for reuse and subsequent reutilization of spent mycosorbents. Acid digestion study suggested adsorption being the primary mechanism accounting for 87% Zn^2+^removal. It was further confirmed by the FE-SEM and EDX analysis. FTIR analysis suggested involvement of amino, hydroxyl, carbonyl, and phosphate functional groups of fungal cell wall in adsorption. The experimental results were in accordance with the tested isotherm and kinetic models, and suggested the role of physical adsorption for Zn^2+^ removal. Noteworthy, the present study showed better sorption capacity in considerably shorter equilibration time compared to previous reports and advocate potential utilization of *A. terreus* SJP02 for bioremediation of Zn^2+^ contaminated wastewater at industrial scale.

**Supplementary Information:**

The online version contains supplementary material available at 10.1038/s41598-025-87749-3.

## Introduction

Water pollution is a major concern for the present industrial world. The major contributor to water pollution includes organic and inorganic pollutants. In India, heavy metals are widespread inorganic pollutants as they are predominantly used by various industries, viz. textile, mining, tannery, dyes, electroplating etc. Although, the government has implemented strict measures for the treatment of wastewater before its disposal in environment, it continues to be prevalent in India. Due to the higher cost of synthetic resins generally used for removing heavy metals, industries frequently discharge their wastewater in the environment without any treatment^1^. Majority of industrial effluent contains high concentration of biologically toxic heavy metals viz. arsenic (As), cadmium (Cd), chromium (Cr), copper (Cu), lead (Pb), mercury (Hg), zinc (Zn), etc^2^. Substantial amount of these heavy metals absorbed by the soil organic matter and retained in the soil. Subsequently, along with water these heavy metals are taken up by plants and enters in the food chain. The majority of the expelled wastewater finally enters and pollute various water sources as well as seep into the soil and underground water^3,4^. The toxicity level of these heavy metals depends on the amount and exposure time to the living beings^5^. However, their prolonged exposure leads to various problems in humans and other organisms. These elements have amendable ability to generate reactive oxygen species (ROS) and further more affect the biological system^6^.

Zinc (Zn) is a heavy metal widely spread in aquatic bodies and soil. Majority of living beings utilizes Zn^2+^ as an essential element for their growth and development^7^. However, excess intake of Zn^2+^ results in adverse health issue in humans as well as plants^1^. In metal form, Zn can cause corrosion of stomach epithelial lining by reacting with the hydrochloric acid present in stomach. Higher concentration of Zn has been reported to cause urinary tract problems. Metal fume fever is one more dangerous disease seen upon zinc exposure which results due to the inhalation of zinc oxide fumes (particle size < 1 mm), usually witnessed in occupational health workers. It leads to various health problems like nausea, soreness in muscle, chest pain, cough etc^7^. As per the recent guidelines of World Health Organization (WHO), drinking water containing Zn at level above 3 mg L^− 1^ may not be acceptable for human consumption^8^. Not only humans, but water bodies including invertebrates also experience oxidative stress as well as altered physiological functions like apoptosis, growth inhibition, immunotoxicity etc. under the excess exposure of Zn^5^. Studies show that rivers contaminated with even less than 2 ppm Zn concentration can affect aquatic lifes by lowering their oxygen carrying capacity and leads to adverse effects^7^. Moreover, noxious Zn levels in soils can adversely affect the growth and metabolic activities in plants by enhanced generation of reactive oxygen species^1^. Hence, there is an urgent need to develop strategies for the removal of excess Zn from industrial wastewater.

There are various chemical and physical methods conventionally used for heavy metal removal including chemical precipitation, adsorption, coagulation, flocculation, ion exchange, membrane separation, etc^9,10^. However, all these methods are expensive, demand huge amount of energy, have low efficiency, produce huge amount of toxic sludge as secondary pollutant, and cannot be used for large scale treatment of heavy metal polluted wastewater^11^. Microbial remediation of heavy metals from wastewater has emerged as an excellent method considering their higher efficiency, cost effectiveness and easy handing in continuous mode^12,13^. Among various microorganisms, in recent years, fungi have emerged as potential candidate for heavy metal remediation as they can survive in adverse environment, have inherent metal tolerance ability and, have greater biosorption and bioaccumulation potential^14,15^. Fungi employ mechanisms viz. chelation of metals on cell wall, precipitation of metal ions by extracellular enzymes and their adsorption, intracellular detoxification by protein binding, active and efflux transporters etc. which play a crucial role in the detoxification and removal of heavy metals^2,16–18^.

Filamentous fungi possess remarkable abilities in the sequestration of metals because of extensive range of functional groups viz. amine, carboxyl, sulfhydryl, phosphate, and hydroxyl) in their cell walls, which act as binding sites for metal pollutants^2,14,19^. Furthermore, due to their filamentous structure, they form ball like pellets during shaking conditions which greatly enhances their efficiency in the sorption process^20^. Filamentous fungal balls (pellets) exhibit a vast range of specific surface area with interconnected spaces, net-like and porous structure that optimizes mass and oxygen transfer, thereby further improving their effectiveness in metal sequestration^21^. Well-adapted fungi isolated from native contaminated soils can be a better source for biosorption of heavy metals an indigenous microbial ecotype results from the long-term adaptation to soil with extreme properties^22^.

The present study examined the Zn^2+^ removal potential of filamentous fungi isolated from the rhizosphere of native plant from metal contaminated industrial area. Based on the maximum Zn^2+^ removal potential in aqueous solution, *Aspergillus terreus* SJP02 was selected for further parametric optimization studies at batch scale. Attempts were also made to investigate the mechanism of Zn^2+^ removal using techniques like acid digestion, field-emission scanning electron microscopy (FE-SEM), energy dispersive X-ray spectroscopy (EDX), fourier transform infrared spectroscopy (FTIR), isotherm & kinetic models. Noteworthy, the present study showed exceptionally high sorption capacity in a noticeably shorter equilibration period. It is the first report on the use of fungal mycelia in “ball form” for bioremediation purposes, which are easier to handle and offer more opportunities for large-scale industrial applications.

## Materials and methods

### Materials

All the chemicals used were of analytical grade and procured from Merck (India) unless otherwise stated. Potato Dextrose Agar (PDA) and Czapek’s Dox Broth media were procured from HiMedia laboratories, Mumbai, India. PCR product purification kit and ITS region primers were purchased from Qiagen (Qiagen, Hilden, Germany) and Sigma-Aldrich (India) respectively. MilliQ water was acquired from Milli-Q Biocel water purification system (Merck KGaA, Darmstadt, Germany).

### Collection of soil samples and isolation of fungi

The soil samples were collected from the industrial contaminated soils near steel products, rubber, spinning mills, graphite dyes, plastic, and welding industries of Mandideep Industrial Area, Bhopal. These industries release large amount of toxic contaminants and heavy metals that harm the ecosystem^23^. The rhizopheric soil samples were collected from naturally grown *Calotropis procera* plants from six spatially separated sites with a minimum of 5 m distance between sampling points. The upper layer of the soil was removed in order to eliminate foreign particles, plant debris, etc. The soil adhering to the plant roots were collected, sealed in plastic bags and placed in the insulated carrier during transport and immediately stored in the refrigerator (4 °C). The collected samples were processed within three days and were sieved using 2 mm mesh before use^24^.

Serial dilution of soil samples was performed followed by plating the inoculum on rose bengal agar medium supplemented with chloramphenicol (30 µg mL^− 1^). The plates were incubated in dark conditions (28 °C) for a period of 4–5 days. Morphologically distinct individual fungal colonies were picked and purified by repeated sub-culturing on PDA to obtain the axenic culture^24^. Glycerol stocks of all morphologically distinct fungi were prepared and stored at -70 °C for further use.

A pooled sub-sample of soil was dried for two days at room temperature, digested using diacid mixture (6 mL HCL: 3 mL HNO_3_) in duplicate and extractants were estimated for heavy metals by Inductively Coupled Plasma-Optical Emission Spectroscopy (ICP-OES, Perkin Elmer Avio 200) analysis^23^. The flow parameters for coolant gas, auxiliary gas, nebulizer gas, and plasma gas were set to 14.0 mL min^− 1^, 0.4 L min^− 1^, 0.70 L min^− 1^ and 10 L min^− 1^, respectively. The nebulizer pressure was 2.4 bar and sample flow rate were 1.5 mL min^− 1^ with a flushing time of 15 s. The wavelength used for Cu, Zn, Pb, Cr, Ni and Co were 327.393, 206.200, 220.353, 267.716 and 228.616 nm, respectively^25^.

### Screening of fungal isolates for Zn2+ removal

All the fungal isolates were screened to check their ability to remove Zn^2+^ from aqueous solution containing 500 ppm Zn^2+^ ion concentration prepared from the stock solution of ZnSO_4_.7H_2_0. The fresh culture of individual fungal isolates was prepared by inoculating them separately in 80 mL of Czapek Dox Broth medium (pH 7.3 *±* 0.2) in 250 mL Erlenmeyer flasks for 5 days at 28 °C on a rotatory shaker (150 rpm) in dark conditions. The mycelial balls were separated from the media by centrifugation (3000 rpm, 4 °C, 10 min.) and washed thrice using autoclaved MilliQ water in order to remove all the traces of media. Typically, 10 g live fungal biomass (fresh weight) was resuspended in 100 mL of Zn^2+^ solution (500 ppm) in 250 mL Erlenmeyer flasks and incubated at 28 °C on a rotatory shaker (150 rpm). Aliquot samples (2 mL) were taken out after 24 h and filtered through 0.02 μm Whatman syringe filters. The filtrate was examined for the remaining Zn^2+^ ion concentration after suitable dilutions using ICP-OES. The experiment was performed in triplicate. The fungal isolate showing maximum Zn^2+^ removal was selected for further experiments.

To compare the biosorption capacity of live and dead biomass, the duly washed live fungal biomass was taken in appropriate amount and autoclaved (121 °C; 15 psi; 20 min.) to prepare the dead biomass^26^.

### Molecular characterization of fungal isolate

Fungal DNA was extracted using CTAB method with minor modifications^27^. Briefly the washed fungal mycelia were mechanically crushed in liquid nitrogen and contents were transferred in microcentrifuge tube containing 500 µL lysis buffer followed by addition 2 µL of RNase, 25 µL of β-mercaptoethanol and incubated at 60 °C in water bath for 1 h. After incubation, 500 µL of PCI mixture (in a ratio of 24 Phenol: 24 Chloroform: 1 Isoamyl alcohol) was mixed in it properly and subsequently centrifuged (10000 rpm, 4 °C, 12 min). 250 µL of the supernatant was taken and properly mixed with 25 µL of 3 M sodium acetate & 250 µL of iso-propyl alcohol, followed by incubation at -20 °C for 1 h in order to precipitate the DNA. Then, the tubes were centrifuged (10000 rpm, 4 °C, 12 min.) and the obtained DNA pellet was washed thrice with 70% ethanol by centrifugation (1000 rpm) for 1 min followed by air drying. Then, the DNA pellet was mixed in minimum amount of MilliQ water and stored at -20 °C until further use.

The fungal isolates were molecularly identified by comparative sequence analysis of internal transcribed spacer (ITS) regions of ribosomal DNA^28^. The ITS regions of ribosomal DNA were amplified using ITS-1 (5’-TCCGTAGGTGAACCTGCGG) and ITS-4 (5’-TCCTCCGCTTATTGATATGC) primers^29^. The Veriti^®^ Thermal Cycler (Applied Biosystems, USA) to carry out the polymerase chain reactions (PCR) in a total volume of 50 µL containing Taq Buffer A (10 mM Tris-HCl, 50 mM KCl and 1.5 mM MgCl_2_, pH 8.3), ITS-1 & ITS-4 primers (50 pmol), deoxynucleoside triphosphates (50 µM), DNA Taq polymerase (1 unit), and genomic DNA template (100–150 ng). The optimized PCR conditions were as follows: initial preheating (94 °C, 2 min), denaturation (94 °C, 1 min), annealing (57 °C, 1.5 min), extension (72 °C, 2 min) as well as a final extension (72 °C, 4 min). The resultant PCR mixtures were purified by HiPurATM PCR product purification spin kit followed by sequencing on ABI prism DNA sequencer (Applied Biosystems, USA). The obtained nucleotide sequences were compared using the basic local alignment search tool (BLAST) network services of National Center for Biotechnology Information (NCBI) database (http://www.ncbi.nlm.nih.gov/). The most closely related species were determined based on Type strain entries^28^. The sequences were deposited in GenBank database to ensure public accessibility, and the accession numbers were acquired. The phylogenetic analysis was conducted utilizing MEGA XI software, wherein a phylogenetic tree was constructed employing the neighbor-joining method with 1000 bootstraps run^30^.

### Parametric optimization for batch and desorption studies

One-factor at a time approach was employed for the optimization of batch experimental parameters^10^. This approach helps study the impact of individual factors without dealing with interactions between multiple variables^31^. The aqueous solution of desired Zn^2+^ concentration was prepared by appropriate dilution of stock solution. The required amount of fungal biomass was exposed to 100 mL of Zn^2+^ solution in 250 mL Erlenmeyer flasks and subjected to different parametric conditions including biomass dosage (1–10 g), contact time (5–240 min), temperature (20–40 ^o^ C), agitation rate (50–250 rpm), pH (5.5-7.0) and initial metal concentration (50–600) (Table 1). After incubation the solution was filtered using Whatman No. 1 filter paper, diluted appropriately, and analyzed for Zn^2+^concentration using ICP-OES (Perkin Elmer, Avio 200). The obtained fungal biomass was dried at 80 °C until constant weight to measure the dry mass. All the experiments were carried out in triplicates.

**Table 1:** Batch experimental parameters used for Zn^2+^ removal by *A. Terreus* SJP02 by taking one-factor at a time approach.

Evaluated parameters	Constant parameters					
	Biomass (FW) (g)	Time (min)	Temperature ( °C)	Agitation rate (rpm)	pH	Metal concentration (mg L^− 1^)
Biomass	1–10	120	28	150	5.65	100
Time	8	5–240	28	150	5.65	100
Temperature	8	60	20–40	150	5.65	100
Agitation rate	8	60	28	50–250	5.65	100
pH	8	60	28	150	5.5–7.0	100
Initial metal concentration	8	60	28	150	5.65	50–600
Desorption studies					Eluant concentration (N)	
Time	8	15–300	28	150	0.01 HCl & HNO_3_	
Eluant concentration	8	120	28	150	0.01–1.5 HCl & HNO_3_	

The sorption capacity (*q*_*e*_) and percentage (%) removal were calculated using Eqs. (1), (2)^32^.

1 $$\:{q}_{e}=\left({C}_{0}-{C}_{i}\right)v∕m$$

2 $$\:\%\:Removal=\frac{{C}_{0}-{C}_{i}}{{C}_{0}}\times\:100$$

Where, *q*_*e*_ represents sorption capacity (mg g^− 1^), *C*_*0*_ and *C*_*i*_ are metal concentration in solution (mg L^− 1^) prior and post sorption process, *v* accounts solution volume (L), and *m* represents biosorbent’s dry mass (g).

### Desorption studies

In order to find out the sorption mechanism, the desorption of Zn^2+^ from fungal biomass surface was carried out using dilute acids. Based on the review of literature, HCl and HNO_3_ were selected to carry out the desorption studies^33^. Initially, the time for desorption was optimized by varying it from 15 to 300 min at constant acid concentration (0.1 N). It was followed by the optimization of eluant concentration (0.01–1.5 N HCl or HNO_3_).

### Acid digestion of fungal biomass

The lyophilized fungal balls i.e. control (without exposure), test (after Zn exposure), and after desorption were used to analyze the sorption (adsorption + absorption) of Zn by fungal cells. Fungal balls were added to 100 mL Erlenmeyer flasks containing tri-acid mixture (1mL HClO_4_: 2 mL H_2_SO_4_: 9mL HNO_3_) and digested on hot plate inside fume-hood untill the development of a clear suspension. After cooling down, the digested suspension was filtered using Whatman no.1 filter paper and the final volume of filtrate was made to 50 mL with Milli-Q water in volumetric flasks. The concentration of Zn ion in the samples was analyzed by ICP-OES. In order to get the actual Zn^2+^ sorption by fungal balls, the amount of Zn^2+^ present in the control fungal balls was subtracted from the test sample (after Zn exposure). The amount of Zn^2+^ after desorption was subtracted from the obtained actual Zn^2+^ sorption value to get the surface adsorbed amount of Zn^2+^. Kumar and Dwivedi^13^ also studied the absorption and adsorption mechanism of Cu^2+^ by *Trichoderma lixii* CR700 using similar calculations.

### FE-SEM and EDX analysis

FE-SEM analysis was performed to determine the changes in the fungal biomass surface post Zn exposure in comparison to untreated biomass. Samples for SEM were prepared by lyophilization of fungal biomass for 48 h followed by gold coating using Quorum Tech Q 150TS Sputter Coater. SEM micrographs were taken by imaging the sample on FEI/Thermo Fischer Apreo LoVac FE-SEM instrument. EDX analysis of samples were carried out using Quorum Tech Q150TS EDX attached with the FE-SEM instrument.

### FTIR analysis

The surface characterization of fungal ball was carried out by FTIR analysis. The untreated fungal biomass (control), Zn treated biomass and biomass after desorption were lyophilized, and diluted with KBr in a ratio of 1:100^13^. FTIR measurements were recorded in the wavenumber range of 400–4000 cm^− 1^ at a resolution of 4 cm^− 1^ on a Frontier FTIR instrument (Perkin Elmer, India).

### Adsorption isotherms and kinetics studies

Langmuir and Freundlich models were used to study the interaction between biosorbent and adsorbate (Zn^2+^) in aqueous solution. Additionally, Temkin and D-R isotherm models were used to analyze the type of adsorption and bonding between the adsorbate and biosorbent. Kinetic models like pseudo-first order and pseudo-second order were applied to understand the correlation in time course data, and were fitted to estimate the rate constant and kinetic parameters^3^. The non-linear forms of these models were fitted with the experimental data using Origin Pro 2022 software.

The monolayer coverage of adsorbate with respect to adsorbent can be explained with the help of Langmuir isotherm model which is based on assumptions that all the adsorption sites are energetically identical and adsorption occur homogenously on the biosorbent’s surface. Langmuir isotherm (non-linear form) is given in Eq. (3).

3 $$\:{q}_{e}=\frac{q\text{m}b{c}_{e}}{1+b{c}_{e}}\:$$

where, *b* is Langmuir isotherm constant (L mg^− 1^) and *C*_*e*_ is equilibrium concentration in the solution (mg L^− 1^).

Langmuir isotherm is characterized by separation parameter at the equilibrium, designated as *R*_*L*_ (dimensionless entity) and expressed by Eq. (4).

4 $$\:{R}_{L}=\frac{1}{1+b{c}_{0}}$$

Where, *R*_*L*_ indicates whether the process of adsorption could be irreversible (R_L_=0), favorable (R_L_<1), linear (R_L_=1) or unfavorable (R_L_>1)^34,35^.

Freundlich isotherm model assumes that sorption process between the sorbate and sorbent is multilayered and heterogenous, and is expressed by Eq. (5).

5 $$\:{q}_{e}={K}_{F}{C}_{e}^{\raisebox{1ex}{$1$}\!\left/\:\!\raisebox{-1ex}{$n$}\right.}$$

Where, *K*_*F*_ represents the Freundlich constant and *n* represents heterogeneity factor for bond distribution^34^.

Temkin isotherm is based on assumption that the heat of adsorption of all molecules in the layer decrease linearly as a result of increase surface coverage and is represented by Eq. (6).

6 $$\:{q}_{e}=B{ln}\left({K}_{T}{C}_{e}\right)$$

Where, *K*_*T*_ is the binding constant at equilibrium and *B* is the heat of sorption (B = RT/*b*). The value of *B* (intercept) and *K*_*T*_ (slope) are obtained from the plot between *q*_*e*_ and *ln C*_*e*_^34^.

Dubinin-Radushkevich (D-R) isotherm is based on assumptions that there is only one type of pore which is uniformly present for the adsorption process and is represented by Eq. (7).

7 $$\:Q={q}_{m}{exp}\left(-k{\epsilon\:}^{2}\right)$$

Where, *ε* is Polanyi potential and is calculated by *RTl*n(1+(1/Ce), *Q* is the amount of metal ions adsorbed (mg g^− 1^), *k* is the adsorption energy (mol^− 2^K^− 1^J^− 1^), and *q*_*m*_ denotes adsorption capacity (mg g^− 1^).

The proportional relationship between rate of solute uptake and gradient in saturation concentration is explained by Pseudo-first order kinetic model which is expressed by Eq. (8).

8 $$\:{q}_{t}={q}_{e}\left(1-{exp}-{k}_{1}t\right)$$

Where, *q*_*t*_ is the amount of metal ions adsorbed (mg g^− 1^) at time *t* and *k*_*1*_ is the rate constant of adsorption (min^− 1^)^34,35^^34,35^.

The kinetic model of Pseudo-second order reaction assumes that the rate limiting step of adsorption process is chemisorption and the non-linear form of equation is expressed by Eq. (9).

9 $$\:{q}_{t}=\frac{{k}_{2}{q}_{e}^{\:\:2}t}{1+{k}_{2}{q}_{t}t}$$

Where, *k*_*2*_ is the adsorption rate constant (g mg^− 1^ min^− 1^)^34,35^.

## Results and discussion

### Heavy metals in rhizopheric soil

The concentration of various heavy metals in the collected rhizospheric soil samples and their WHO permissible limits in contaminated soil are shown in Supplementary Table S1. In general, almost all the tested heavy metals were found to be present in elevated concentration, surpassing the permissible limit set by WHO for contaminated soils. Particularly, the concentration of Cu (1073 *±* 0.7 mg Kg^− 1^) and Zn (1015.3 *±* 1.4 mg Kg^− 1^) were found to be drastically higher (> 20 time). These results were in accordance with Rathor et al.^23^ who reported significantly higher concentration of Cu and Zn in the soils of Mandideep industrial area. Moreover, the concentration of other heavy metals viz. Co, Ni, and Cr was also found to be much higher than the maximum permissible limits, which highlight that the Mandideep industrial area soils are highly polluted with heavy metals.

### Screening of fungal isolates and molecular characterization of isolate showing maximum Zn2+ removal

A total of 15 fungal isolates (SJP01 to SJP15) were chosen based on distinct morphology, and purified further, followed by their glycerol stocks preparations. All these 15 fungal isolates were screened for their Zn^2+^ removal efficiency from aqueous solution (Supplementary Table S2). The maximum percent Zn^2+^ removal (25.86%) was recorded for isolate SJP02 followed by SJP03 (23.04%). Whereas, the least Zn^2+^ removal efficiency was shown by isolate SJP11 (3.28%). On the basis of its maximum Zn^2+^ removal ability, isolate SJP02 was selected for detailed molecular characterization. Based on the comparative sequence analysis of ITS region of ribosomal DNA, it was identified as *Aspergillus terreus* SJP02. The ITS1-5.8 S-ITS2 gene complex sequence was duly submitted to NCBI GenBank database with the accession number OR726084. The fungal isolate has also been deposited in the Microbial Type Culture Collection at the Institute of Microbial Technology (IMTech), Chandigarh, India and is available at the public domain with the MTCC number 13,417.

The phylogenetic tree (Supplementary Fig. S1) depicts the evolutionary relationship of *Aspergillus terreus* isolate SJP02 with other closely related reference isolates. It revealed that isolate SJP02 showed close similarity (100% identity) with *Aspergillus terreus* ATCC1012 and clustered together in the phylogenetic tree. *Aspergillus neoterreus* CGMCC 3.20891, *Aspergillus terreus var. subfloccosus* CBS 117.37, and *Aspergillus alabamensis* CBS 125,693 were also sub-clustered with it and had identity of above 95% in GenBank. However, it was found to be notably segregated from the remaining members of genus *Aspergillus*, belonging to division *Ascomycota*. Multiple reports showed prevalence of fungi belonging to *Ascomycota* division in the soils contaminated with heavy metals as well as their potential in the removal of heavy metals^36–38^. Very recently, AbdelGalil et al.^39^ also reported the potential of *Aspergillus terreus* for biosorption of Sr (II) and Y (III) from radioactive waste.

### Biosorption capacity of live and dead biomass for Zn2+ removal

Live fungal biomass showed higher sorption capacity (*q*) of 10.7 mg g^− 1^ in comparison to the dead biomass (9.5 mg g^− 1^) (Supplementary Table S3). Multiple reports showed that live cells have better heavy metal removal efficiency than dead cells^40,41^. Chen et al.^42^ conducted a comprehensive investigation on *Penicillium simplicissimum*, and compared the efficacy of live and dead cells to remove various metal ions, including Cd, Cu, Pb, Zn, and Cr (III). Their findings revealed that live cells demonstrated superior metal uptake in comparison to dead cells. The higher metal uptake in live fungal cells could be attributed to the combined mechanisms of bioaccumulation and surface adsorption. On contrary, the metal removal by dead cells is primarily driven only by the surface adsorption. Ahmed ElGendy et al.^43^ investigated the potential of *Cladosporium sp.* NRCA8 dead biomass to remove multiple heavy metals and reported a significantly low Zn sorption capacity (1.70 mg g^− 1^) when tested with 100 mg L^− 1^ of initial Zn concentration. When the metal ions enter the live cell, they may be sequestered by binding with chelators like metallothionein, glutathione or get compartmentalized within vacuoles or get transformed into less toxic form by enzymes within the cells, whereas the surface adsorption could be coupled with precipitation in some instances^13,42,44^.

### Parametric optimization

#### Effect of biosorbent dosage

**Fig. 1:**
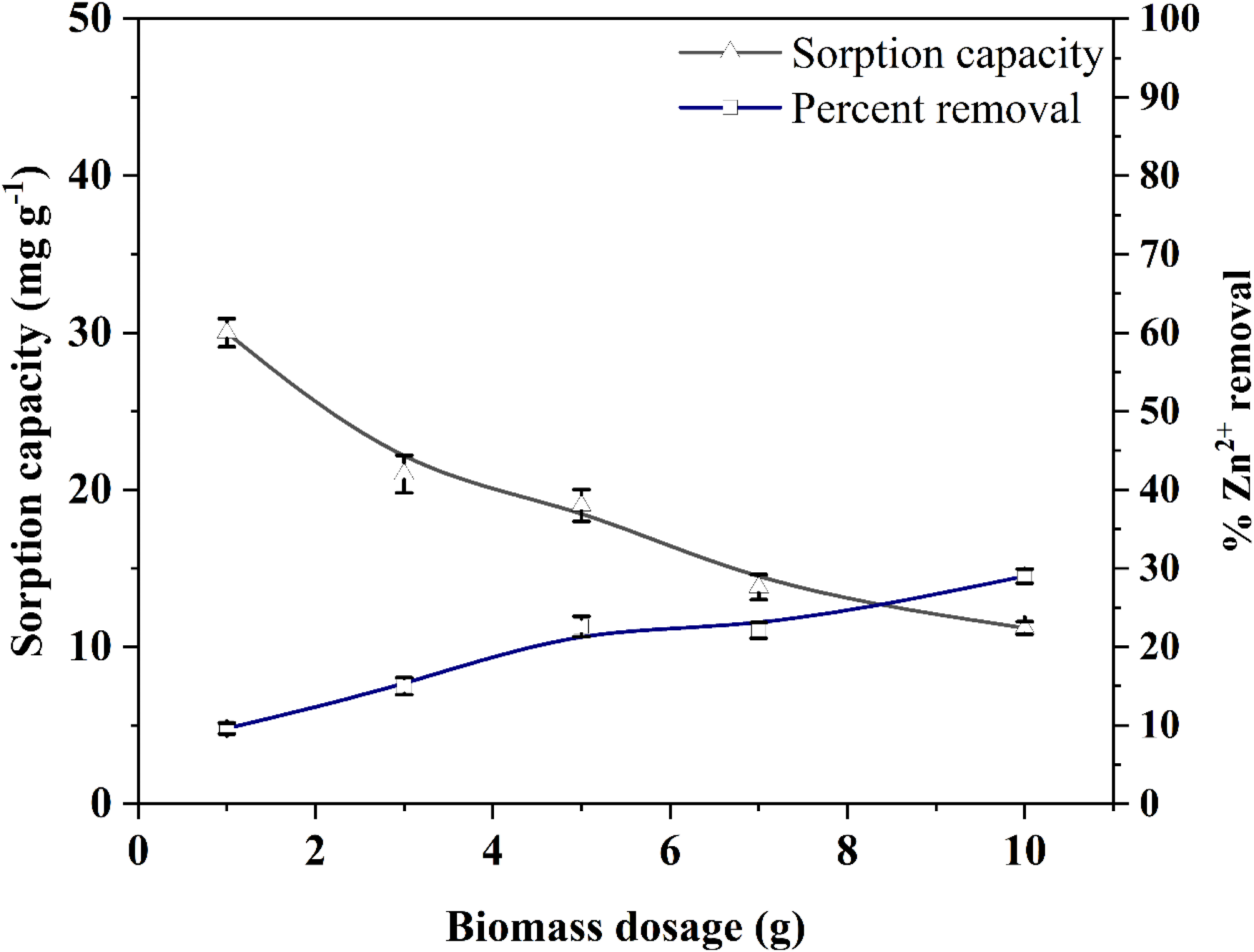
Effect of biomass dosage on Zn^2+^ removal from aqueous solution by *A. terreus* SJP02. Bars represent the “standard deviation.”

The quantity of biosorbent is a crucial factor in the sorption process. Figure 1. represents the effect of different biomass dosage on Zn^2+^ removal efficiency and sorption capacity of *A. terreus* SJP02 biomass. A gradual increase in the percentage Zn^2+^ removal from 9.6 *±* 0.7 to 29 *±* 0.9% was observed, while the sorption capacity was found to decrease from 30 *±* 0.9 to 11.2 *±* 0.4 mg g^− 1^ with the increase in wet biomass. The observed increase in percentage removal with the sorbent dosage attributed to the availability of large number of active sites^45,46^. However, the greater amount of active sites for the same quantity of sorbate molecules could be the reason for the decrease in sorption capacity^41^. In general, the optimum dose of biosorbent is typically determined by the intersection point of sorption capacity and percentage removal graph^41,47,48^. From the intersection point of sorption capacity and percentage removal, the optimum value of sorbent dosage was determined as ~ 8 g of wet biomass (Fig. 1). Hence, further studies were conductedusing 8 g of wet biomass which was equivalent to 0.184 g dry weight.

#### Effect of contact time

**Fig. 2:**
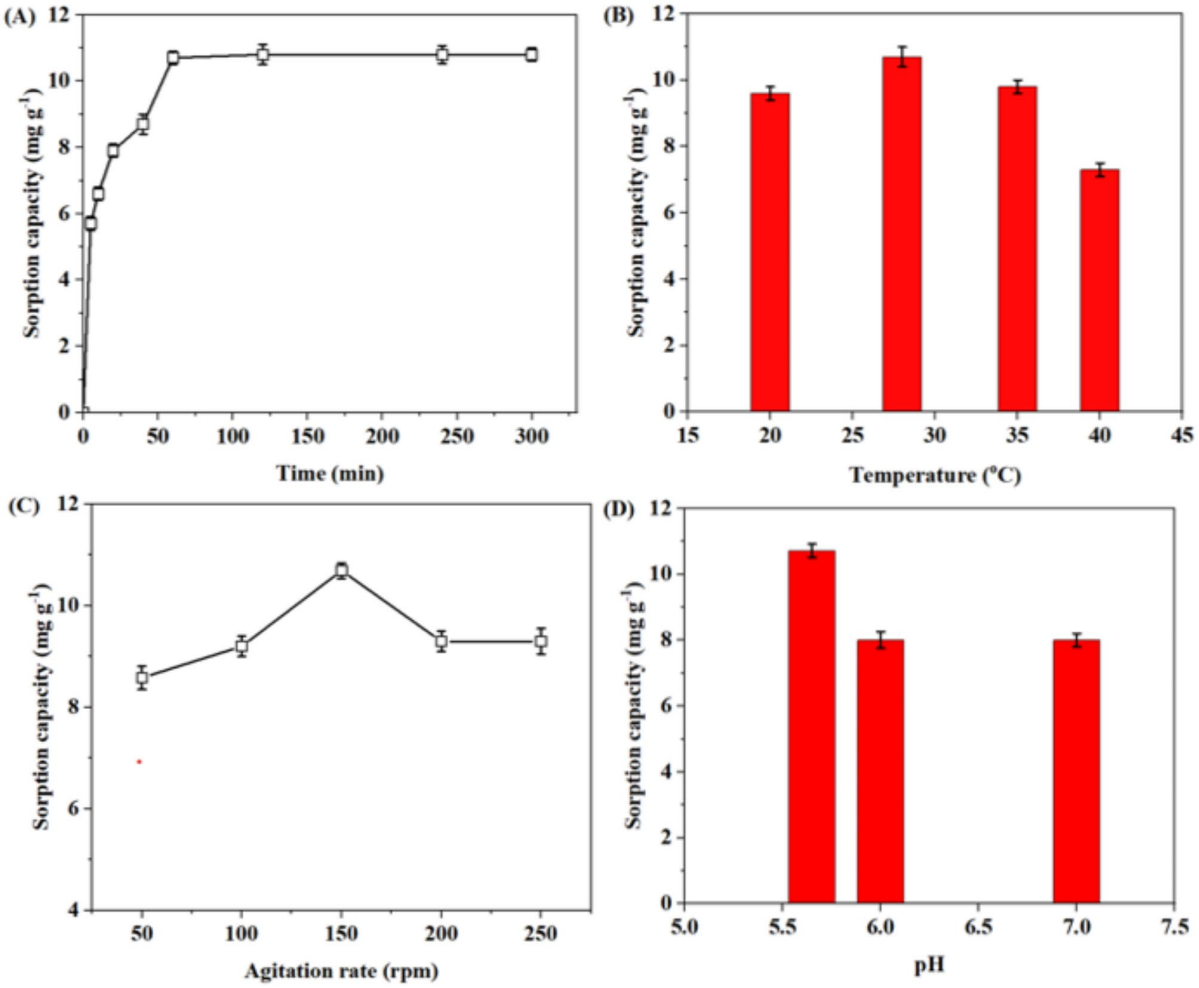
Effect of (**A**) contact time and (**B**( temperature (**C**) agitation rate and (**D**) solution pH on Zn^2+^ removal from aqueous solution by *A. terreus* SJP02. Bars represent the “standard deviation.

Contact time is a critical component that governs the sorption process’s rate kinetics. Figure 2A represents the change in Zn^2+^ sorption capacity of *A. terreus* SJP02 at various time intervals. The time required to attain the equilibrium sorption capacity (10.7 *±* 0.2 mg g^− 1^) was found to be 60 min for 100 mg L^− 1^ initial Zn^2+^ concentration, after which no further increase in the sorption capacity was observed. It may be due to the saturation of active sites by Zn^2+^ at the sorbent surface^49^. The sharp increase observed in the curve at the beginning of the contract period can be attributable to the high adsorption rate and indicated that Zn^2+^ was favorably sorbed by the fungal biomass^3^. In a similar study, Li et al.^26^ demonstrated the sorption of zinc using the live and dead cells of *Streptomyces ciscaucasicus* strain CCNWHX 72 − 14 at varying time intervals. However, the equilibrium time for sorption of Zn^2+^ reported in their study was 7–8 h which is quite higher in comparison to the equilibrium time of 1 h in the present study and suggest the better sorption potential of *A. terreus* SJP02.

#### Effect of temperature

Temperature is always considered an important factor to scale up the biosorption process. The effect of temperature range (20–40 °C) on Zn^2+^ removal is shown in Fig. 2B. Because of active site binding of Zn^2+^ on the biomass, the fungus at the temperature of 28 °C, where it typically grows the maximum Zn^2+^ sorption capacity of 10.7 *±* 0.3 mg g^− 1^ was found at 28 °C which is the well known optimum temperature required by the fungi for their growth^50^. However, relatively lower Zn^2+^ sorption capacity was observed at lower (20 °C) and higher temperatures (35 °C & 40 °C). The lower and higher temperatures than the optimal growth temperatures have been reported to affect the enzymatic activity in metabolic processes and fungal biomass growth^17^. Moreover, temperatures higher than 28 °C could distort the binding sites on the fungal biomass surface, thereby results in decreased sorption capacity^41^. The elevated temperature may also hamper the cell wall configuration, membrane’s integrity, functional group’s ionization (present in the cell wall) and decrease absorbate’s thermal energy, thus ultimately lowering the removal efficiency^17,51,52^.

#### Effect of agitation rate

The mycoremediation process depends on the effective interactions between the sorbate and sorbent, and the agitation rate play a crucial role for these interactions. Figure 2C illustrates the impact of agitation rate (rpm) on the Zn^2+^ removal by *A. terreus* SJP02. The maximum sorption capacity of 10.69 *±* 0.15 mg g^− 1^ was observed at an agitation rate of 150 rpm, which may be due to the optimum interactions between fungal biomass and Zn^2+^ solution that enhanced the rate of Zn^2+^ transfer from solution to sorbent surface. Lower (50 and 1000 rpm) and higher (200 and 250 rpm) agitation rates resulted in significantly less sorption capacities in comparison to 150 rpm. At lower agitation rates, the exposure between the sorbent and sorbate (Zn^2+^) might be improper, whereas higher agitation rates have been reported to cause vortexing effect which do not allow sufficient contact time between the sorbent and sorbate^41^. Moreover, at higher agitation rate, the reduction in the sorption capacity may be attributed to the desorption of Zn^2+^ or the hindrance in the fungal biomass growth by physical damages^17^. Previous studies for sorption of metal ions using fungus also reported the optimum agitation rate around 150 rpm^40,53^. Hence, the optimum agitation rate was considered as 150 rpm.

#### Effect of pH

Figure 2D represents the effect of solution pH on Zn^2+^ removal by *A. terreus* SJP02. It is well known that the optimal pH range for maximum fungal growth is typically between 5.5 and 6.5^54^. pH values below 5.5 was not evaluated since lower pH levels can lead to the competition between protons and non-specific binding of Zn^2+^ on active sites, which would have resulted in decreased sorption capacity^41^. Whereas, pH range above 7.0 was not tested as it could lead to precipitation of Zn^2+^ ions as zinc hydroxide. Change in the solution pH showed significant effect on Zn^2+^ sorption by *A. terreus* SJP02. The highest sorption capacity of 10.71 *±* 0.2 mg g^− 1^ was observed in case of ZnSO_4_.7H_2_O solution with Zn^2+^ concentration of 100 mg L^− 1^ at a natural pH of 5.65. A significant decrease in the sorption capacity was noticed with the increase in pH values. At high pH, hydronium ions dissociate, allowing positively charged metal ions to bind to negatively charged microbial surfaces^55^. The observed optimum pH for Zn^2+^ removal to be is in agreement with previous reports^40,42^.

Moreover, after completion of pH experiment, the relative growth of *A. terreus* SJP02 was measured on PDA plates (pH 5.62) in order to prove that whether the fungus survives after exposure to different pH conditions. Fungal biomass exposed to zinc solution at natural pH 5.65 exhibited maximum growth with a diameter of 3.15 cm. Whereas, significantly lesser growth i.e. 2.55 and 2.45 cm diameter was observed at pH 6.0 and 7.0, respectively. These results suggests that at pH 6.0 and 7.0, there was the fungal biomass experienced lesser metabolic activity which resulted in slow growth in lag phase. Therefore, zinc solution with natural pH 5.65 was considered optimum.

#### Effect of the initial concentration of Zn2+

**Fig. 3:**
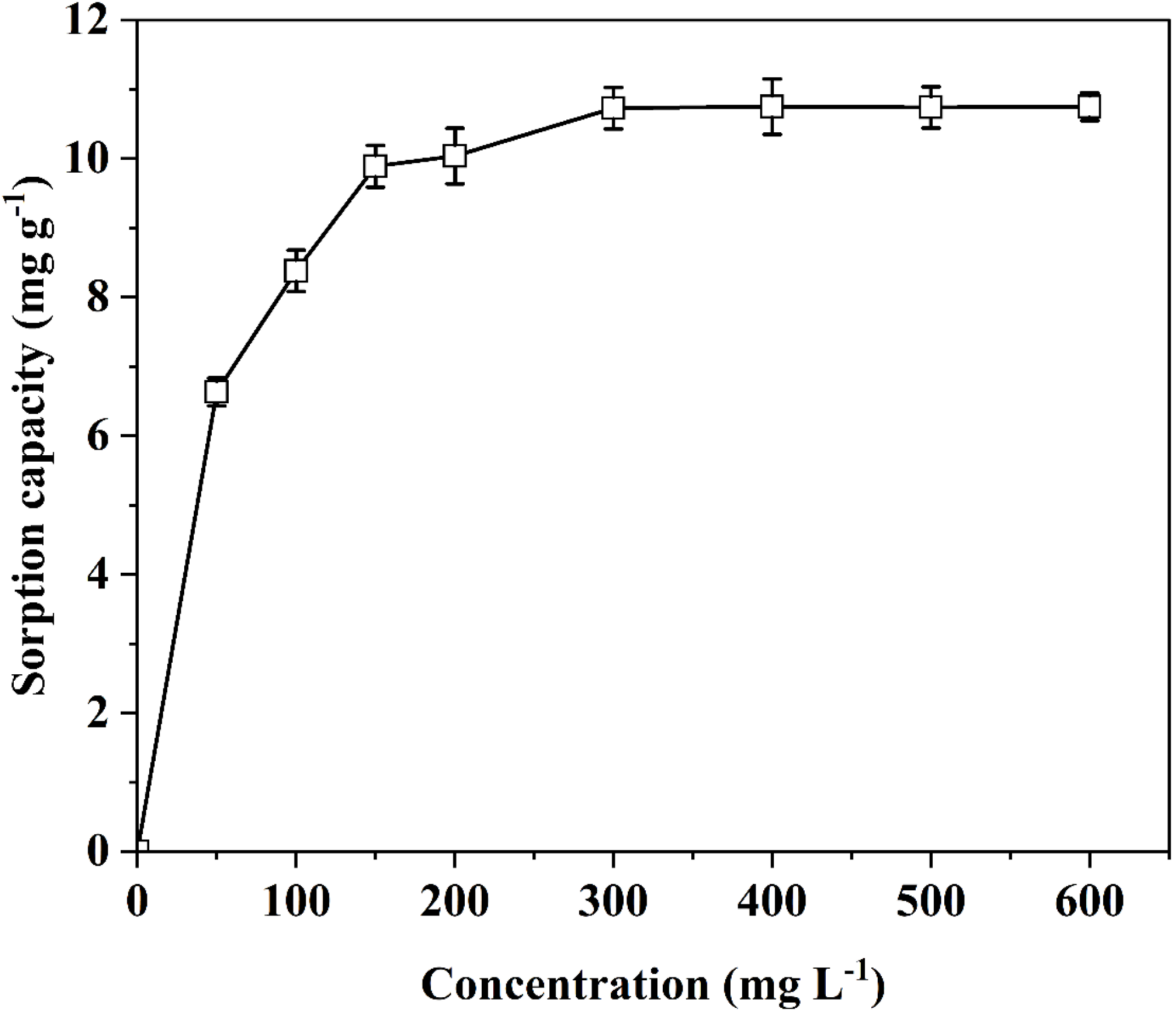
Effect of initial concentration of Zn^2+^ on the sorption capacity of *A. terreus* SJP02. Bars represent the “standard deviation.”

The effect of the initial Zn^2+^concentration of on the sorption capacity of *A. terreus* SJP02 is shown in Fig. 3. The sorption capacity was found to increase from 6.63 *±* 0.2 to 10.73 *±* 0.2 mg g^− 1^ with the increase in the initial concentration of Zn^2+^ from 50 to 300 mg L^− 1^, respectively. However, no significant change was observed in sorption capacity with the further increase in the initial concentration of Zn^2+^ up to 600 mg L^− 1^. It may be due to the exhaustion of a fixed number of active sites available in the fungal biomass which would have resulted in the saturation of sorption capacity at 300 mg L^− 1^ of Zn^2+^ concentration. El Sayed et al.^56^ reasoned the maximum adsorption capacity at a Zn(II) concentration of 600 mg L^− 1^ can be attributed to enhanced mass transfer, increased kinetic energy, and a higher availability of metal ions, which in turn increases the likelihood of collisions between the biosorbent and the metal ions.

### Desorption studies

**Fig. 4:**
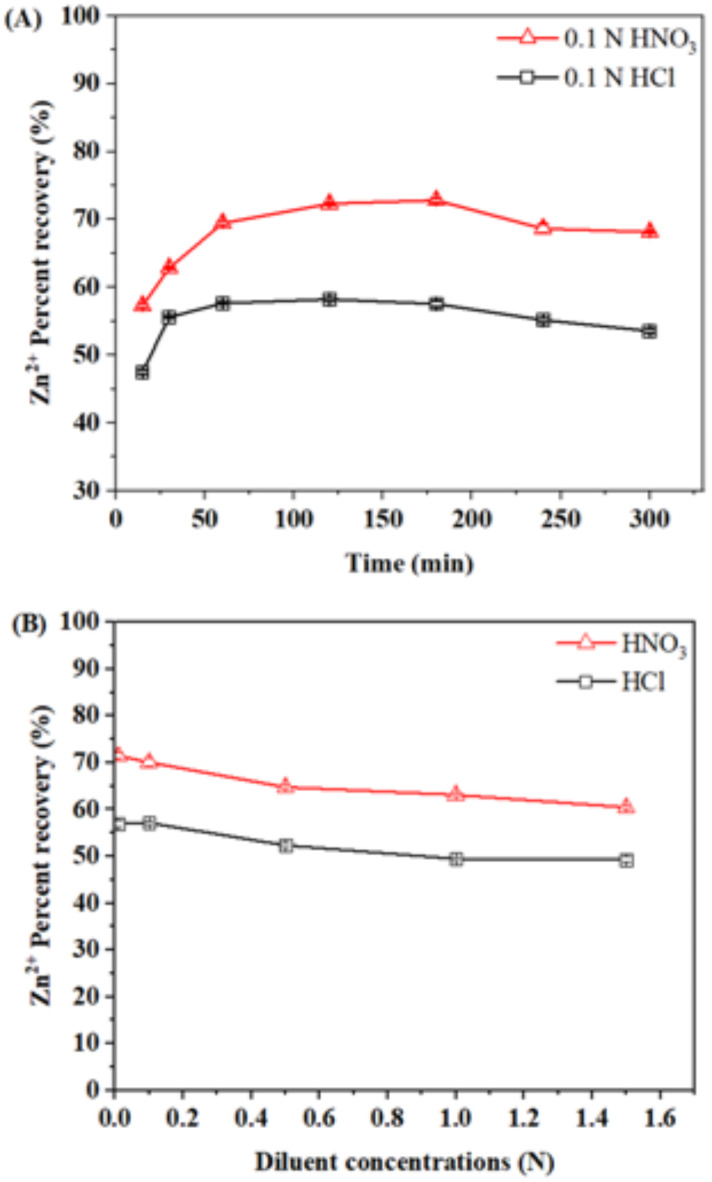
Desorption of Zn^2+^ (**A**) at various time intervals (15–300 min) and (**B**) at different concentrations of HNO_3_ and HCl (0.01–1.5 N) at 120 min. Bars represent the “standard deviation.”

Desorption studies were conducted to explore the reusability efficacy of spent biosorbent. The percentage of Zn^2+^ desorption ranged from 57.26 *±* 0.3% to 68.13 *±* 0.2% for 0.1 N HNO_3_ and 47.47 *±* 0.3% to 58.54 *±* 0.3% for 0.1 N HCL at time interval from 15 to 300 min, respectively (Fig. 4A). The highest percent recovery was obtained as 72.31 *±* 0.2% for 0.1 N HNO_3_ and 58.20 *±* 0.2% for 0.1 N HCL at 120 min. Hence, the optimum desorption time was considered to be 120 min for both these desorbing agents.

Further, the concentration of both desorbing agents was optimized by performing the desorption experiments using Zn^2+^ loaded biomass at different concentrations (0.01 N, 0.1 N, 0.5 N, 1 N, 1.5 N) of HNO_3_ and HCl (Fig. 4B). The percent recovery was found to decrease from 71.46 *±* 0.56% to 60.46 ± 0.45% for HNO_3_ and 56.99 ± 0.7% to 49.30 ± 0.59% for HCl with an increase in the concentration from 0.01 to 1.5 N, respectively. The obtained results showed that 0.01 N HNO_3_ exhibited better percent recovery of Zn^2+^ (71.46%) within 120 min in comparison to 0.01 N HCL (56.99%). Thus, 0.01 N HNO_3_ and 120 min were selected as the optimum concentration and time respectively, for the desorption of Zn^2+^ loaded *A. terreus* SJP02. Similarly, Igberase et al.^57^ reported a desorption efficiency of 79.6% by using 0.5 M HNO_3_ for Cu^2+^ removal by polyaniline grated chitosan beads.

### Mechanism of Zn2+ sorption

#### Acid digestion studies

The acid digestion studies were performed to find out the underlying mechanism of Zn^2+^ removal by *Aspergillus terreus* SJP02. The two major mechanisms could be adsorption and/or absorption. The primary mechanism for Zn^2+^ removal was found to be fungal cell wall surface adsorption (0.754 − 0.098 = 0.656 mg) which accounted for 87% of Zn^2+^ removal (Table 2). Whereas, intracellular absorption of Zn^2+^ (0.098 mg) had a comparatively minor effect and accounted only for ~ 13% Zn^2+^ removal. Sorption of heavy metal ions onto the cell wall of fungal biomass has been reported in multiple previous reports and majority of them showed involvement of the surface functional groups for the adsorption of metal ions^13,58,59^. The intracellular absorption of Zn^2+^ has been reported to be facilitated by membrane transporters, followed by which it may bind with metal chelating proteins^44,58^. Further, it may get compartmentalized by the help of ZRT (zinc-regulated transporters), ABC (ATP-binding cassette) transporters, as well as CDF (cation diffusor facilitator) family of proteins^2,51^.

**Table 2:** Concentration of adsorbed and absorbed zn by *A. terreus* SJP02.

Total Zn^2+^ in control fungal biomass (mg)	Total Zn^2+^ in test fungal biomass after sorption experiment (mg)	Actual Zn^2+^ sorbed by test fungal biomass (mg)	Total Zn^2+^ in test fungal biomass after desorption experiment (mg)	Total zinc after desorption experiment (mg)
0.014 + 0.05	0.768 + 0.14	0.754 + 0.08	0.112 + 0.07	0.098 + 0.09

#### FE-SEM analysis

**Fig. 5:**
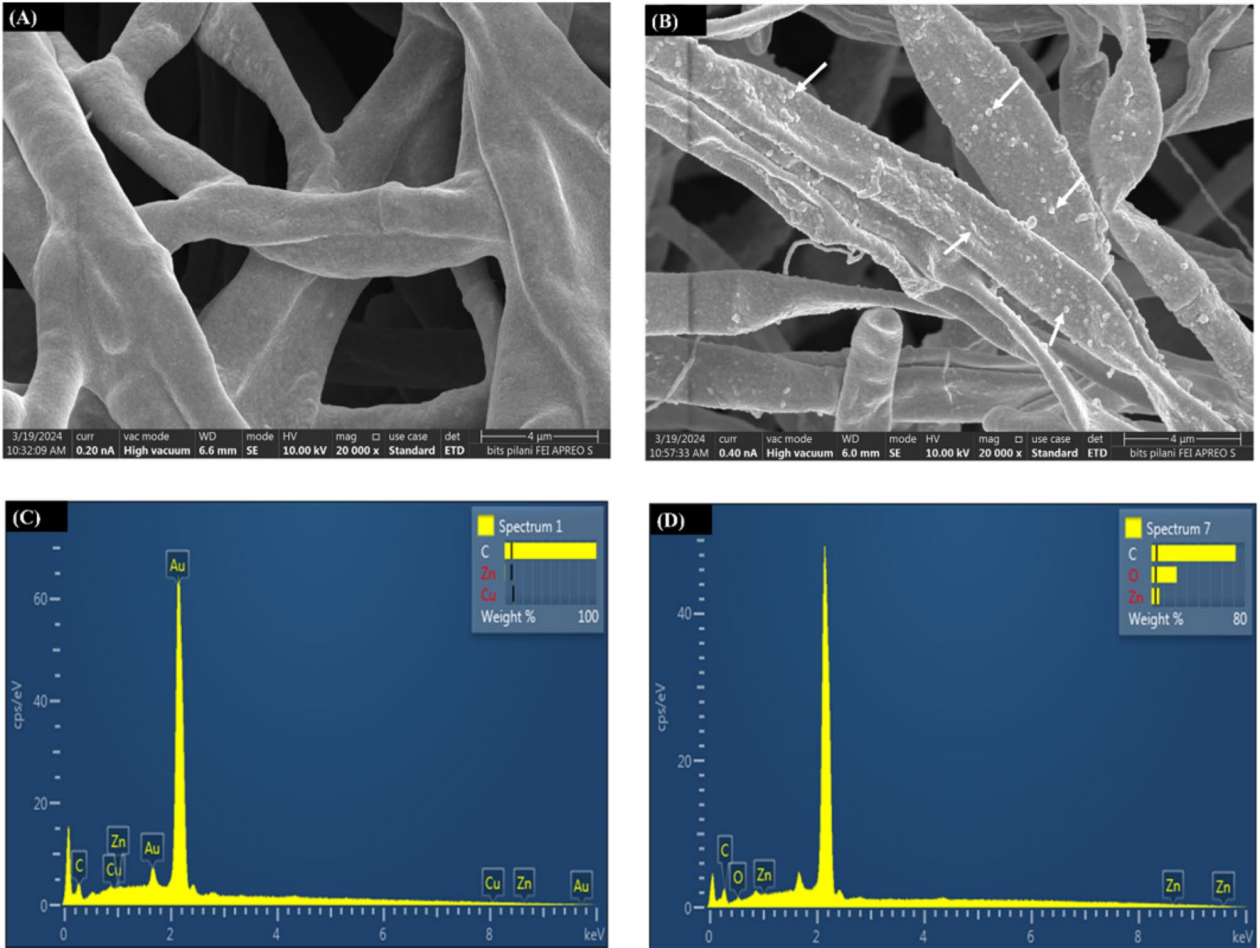
FE-SEM micrographs showing (**A**) untreated and (**B**) Zn treated biomass of *A. terreus* SJP02. EDX spectrum depicting elemental composition of (**C**) untreated and (**D**) Zn treated biomass of *A. terreus* SJP02.

FE-SEM analysis was performed to examine the deposition of zinc on fungal biomass surface. In comparison to the control biomass (Fig. 5A), the treated fungal biomass surface clearly depicted the deposition of zinc on its surface (Fig. 5B inset). Moreover, the mycelia were observed to be constricted and aggregated in comparison to the control biomass. Such morphological changes are frequently observed adaptive metal tolerance behavior in fungi^60^. Fungi are known to show a variety of morphological responses under heavy metal toxicity which include production of extracellular polymeric substances (EPS), change in mycelia colour, and reducing its surface area by constricted, elongated and aggregating mycelia^13,61,62^.

EDX analysis showed significantly higher atomic Zn content (1.31%) in zinc treated biomass (Fig. 5D) in comparison to control (Fig. 5C) which showed only 0.02% Zn content (Supplementary Table S4). It suggests that during sorption process, Zn^2+^ ions was primarily deposited onto the fungal mycelia, aiding in the removal process. However, the distribution pattern of Zn^2+^ adsorption was observed to be non-uniform at various sites of fungal mycelia surface. The EDX analysis of fungal biomass after desorption showed absence of Zn on the biomass surface (Supplementary Table S4).

#### FTIR analysis

**Fig. 6:**
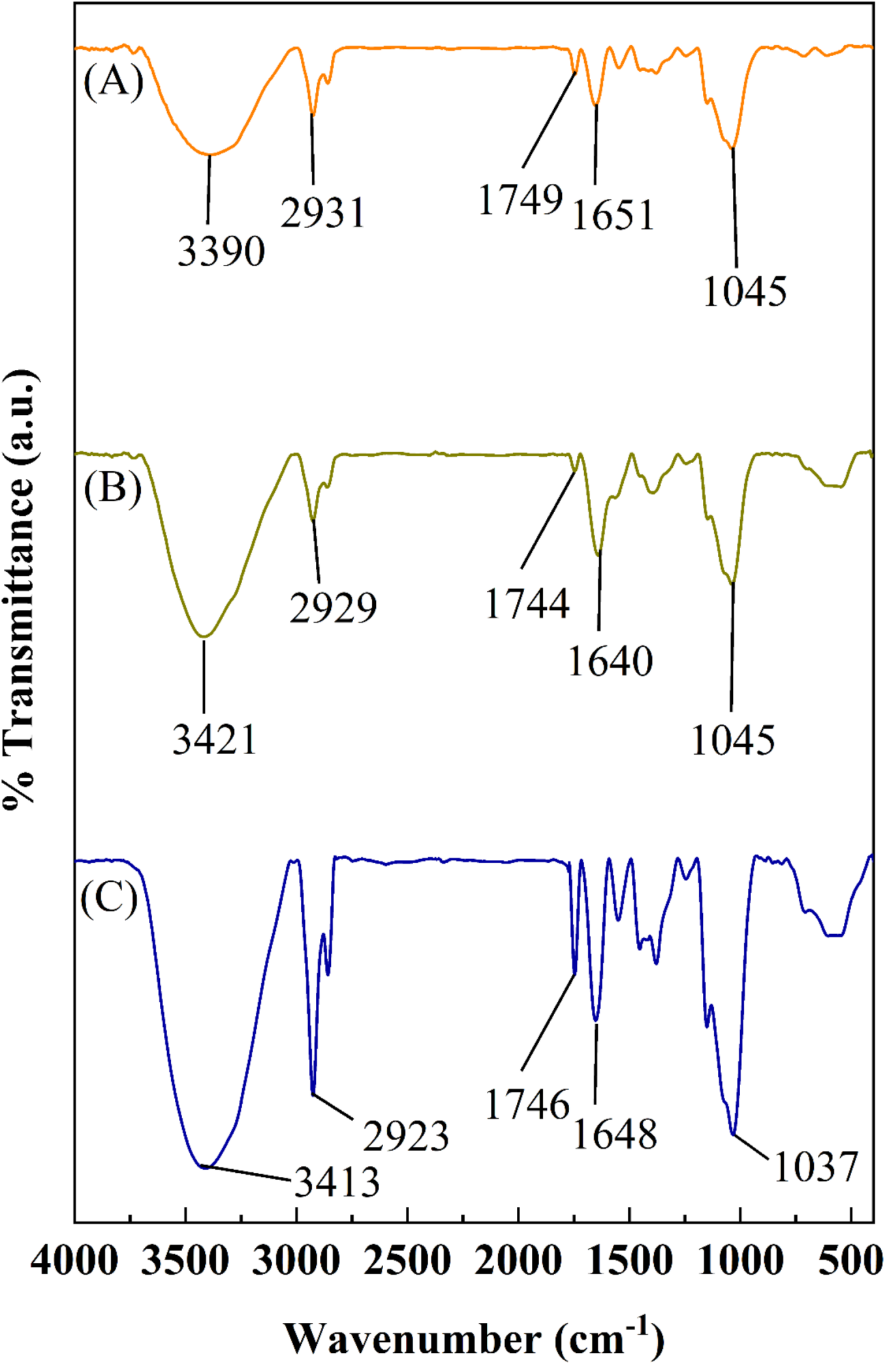
FTIR spectra of *A. terreus* SJP02 (**A**) untreated biomass (**B**) biomass after sorption of Zn^2+^ (**C**) biomass after desorption of Zn^2+^.

The changes in FTIR spectra of *A. terreus* SJP02 biomass surface after sorption and desorption of Zn²⁺ were compared with the untreated biomass (control) to identify the surface functional groups involved in the sorption of Zn²⁺ (Fig. 6). A broad peak observed at 3390 cm⁻¹ corresponding to N-H and O-H stretching vibrations in the control biomass was found to be shifted to 3421 cm⁻¹ after sorption of Zn²⁺. After desorption of Zn²⁺ from biomass surface, this peak again shifted to 3413 cm⁻¹. Similar characteristic peak shifting pattern were reported by AbdelGalil et al.^39^ while studying the surface functional groups responsible for the removal of Sr (II) and Y(III) by *Aspergillus terreus*. The sharp peak at 2931 cm^− 1^ in the control biomass corresponds to the asymmetric stretching vibrations of CH_2_ functional group^63^. After sorption of Zn²⁺on fungal biomass, it shifted to 2929 cm⁻¹ and after desorption of Zn²⁺, it shifted to 2923 cm^− 1^.

After sorption and desorption of Zn²⁺, the 1749 cm^− 1^ peak (in control) shifted at 1744 cm^− 1^ & 1746 cm^− 1^, respectively, which correspond to C = O stretching vibrations of ester. Moreover, there was a noticeable increase in the intensity of peak at 1746 cm^− 1^ after desorption of Zn²⁺ from biomass surface. Chen et al.^58^ also reported the shifting of C = O stretching vibration peak while studying the biosorption of heavy metal ions by *Penicillium simplicissimum*. The peak at 1651 cm^− 1^ (control biomass) was observed shifted to 1640 cm^− 1^ and 1648 cm^− 1^ after sorption and desorption of Zn²⁺, respectively, with significant increase in the peak intensity. These peaks attributed to the C = O stretching mode of carbonyl group as reported by El Sayed et al.^56^ while studying the removal of zinc ions using *Fusarium solani*.

No change in the peak at 1045 cm^− 1^ was observed after the sorption of Zn²⁺. However, this peak shifted to 1037 cm^− 1^ after the desorption of Zn²⁺. Peaks ranging between 1045 cm^− 1^ to 1035 cm^− 1^ corresponds to orthophosphate groups present in the glycoproteins of fungal cell membrane, which carry a negative charge at pH levels above 3.0 and has been reported to interact with the positively charged Zn²⁺ and other cations^64^. Overall, the FTIR analysis suggested the presence of amino (N-H), hydroxyl (OH^−^), carbonyl group (C = O), and phosphate functional groups in *A. terreus* SJP02 which facilitated the binding of Zn²⁺ through electrostatic interactions. The obtained results were in agreement with Mushtaq et al.^65^, who utilized *Trichoderma* sp. isolated from tannery waste for the removal of various heavy metal ions. Fungal cell walls have complex structures made up of glucan, chitin, and glycoproteins with different functional groups, which are reported to be responsible for the sorption of heavy metal ions^66,67^.

#### Equilibrium isotherm

**Fig. 7:**
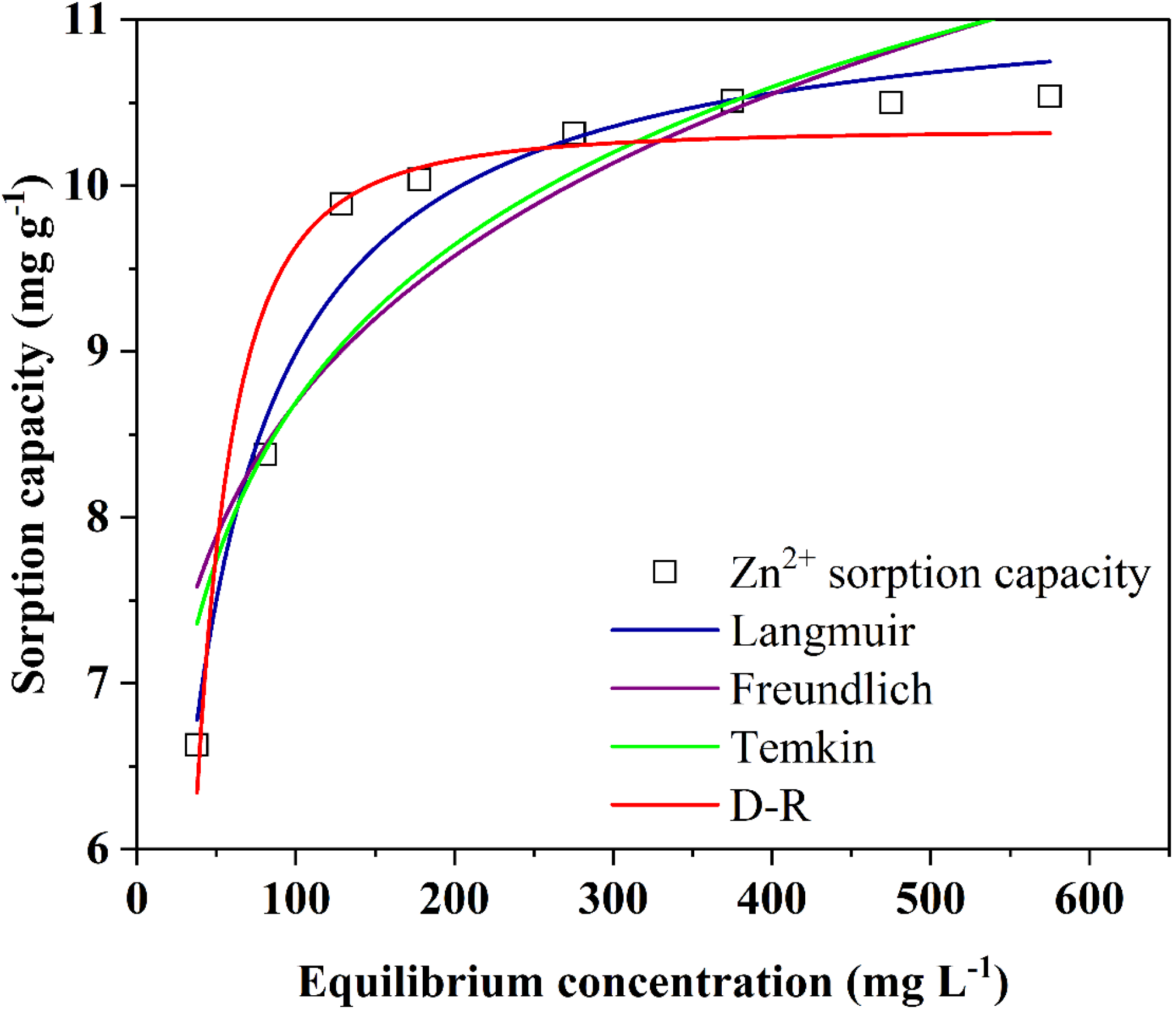
Isotherm models for Zn^2+^ removal in aqueous solution by *A. terreus* SJP02.

The values obtained from the optimization studies were fitted using equilibrium isotherm models (Fig. 7). The Langmuir and Freundlich isotherms are commonly used to analyze the equilibrium behavior of the sorption process, while the nature of the sorption process is analyzed using the Temkin and Dubinin-Radushkevich (D-R) models^65,68^. The coefficient of determination (*r*^2^) values were determined by fitting the non-linear form of isotherm equations with the experimental equilibrium data, and the parameters obtained are depicted in Table 3. The Langmuir isotherm exhibited a higher *r*^2^ value of 0.97 in comparison to the Freundlich isotherm model (0.81) for Zn^2+^ removal which advocated the suitability of Langmuir isotherm model for adsorption of Zn^2+^ by fungal biomass. It suggest that coverage of Zn^2+^ on the *A. terreus* SJP02 fungal biomass surface occurred in a monolayer homogenously^69^. Langmuir isotherm model predicted maximum sorption capacity of 11.21 mg g^− 1^, which was quite close to the obtained experimental sorption capacity i.e. 10.73 mg g^− 1^ (biomass dose: 8 g; contact time: 60 min; temperature: 28 ºC; pH: 5.65; initial Zn^2+^concentration: 300 mg L^− 1^). While investigating Cu^2+^ adsorption using co-integrated *Aspergillus flavus* ZJ-1 and *Chlorella vulgaris* WZ-1, based on higher *r*^2^ value, Zhang et al.^69^ also reported suitability of Langmuir isotherm in comparison to the Freundlich isotherm model. Langmuir constant, ‘*b’*, was utilized to calculate the *R*_L_ values, which explain the feasibility of the sorption process. The *R*_*L*_ values between 0 and 1 indicate that adsorption is favorable and *R*_*L*_ values more than 1 suggest that adsorption is unfavorable^70^. In present study, the *R*_*L*_ values ranged from 0.33 − 0.04, which confirmed favorable sorption of Zn^2+^ on fungal biomass surface. It also supports that the Langmuir model accurately describes the adsorption process. Asha et al.^71^ also described that *R*_*L*_ value provides insight into the nature of the isotherm curve and indicate the type of adsorption behavior which can be irreversible (*R*_*L*_ = 0), favorable (0 < *R*_*L*_ < 1), linear (*R*_*L*_ = 1), or unfavorable (*R*_*L*_ > 1).

**Table 3:** List of parameters evaluated and regression obtained from the fitting of different models.

Isotherms			
Model	Parameters	Values	Regression coefficient (*r*^2^)
Langmuir	*Q*_m_ (mg g^− 1^)	11.21 *±* 0.25	0.97
	*b* (L mg^− 1^)	0.04 *±* 0.14	
	*R*_L_	0.33 − 0.04	
Freundlich	*n*_F_	7.14	0.81
	*K*_f_ (mg g^− 1^) (dm^3^ mg^− 1^)^1/n^	4.56 *±* 0.17	
Temkin	*K*_T_ (L min^− 1^)	1.37 *±* 0.23	0.85
	*B* (J mol^− 1^)	5.67 *±* 0.15	
	*b*_T_	441.57	
D-R	*Q*_m_ (mg g^− 1^)	10.34 *±* 0.19	0.92
	*K* (mol^2^ kJ^− 2^)	1.14E-04	
	*E* (KJ mol^− 1^)	0.66	
Kinetic models			
Pseudo first order	*q*_e_ (mg g^− 1^)	10.27 *±* 0.18	0.93
	*k*_1_ (min^− 1^)	0.10 *±* 0.01	
Pseudo second order	*q*_e_ (mg g^− 1^)	11.01 *±* 0.3	0.97
	*k*_2_ (g mg^− 1^ min^− 1^)	0.01	

The obtained values of *K*_*f*_ (4.56 (mg g^− 1^) (dm^3^ mg^− 1^)^1/n^) and *n*_*F*_ (7.14) from Freundlich model were found to be more than 1, which demonstrated favorable sorption of Zn^2+^ onto the surface of *A. terreus* SJP02. Freundlich isotherm describes adsorption on heterogeneous surfaces, where adsorption sites have different affinities^72^. According to Allahyar and Özeroğlu (2021)^73^, the value of *n*_*F*_ indicate the nature of adsorption. If *n*_F_ > 1, the adsorption process is physical, while a value between 0 and 1 suggests chemical adsorption. Additionally, *n*_*F*_ provides insight into the surface heterogeneity of the adsorbent, with values closer to zero indicating greater heterogeneity. While studying the sorption of zinc on the live cells of *Streptomyces ciscaucasicus*, Li et al.^26^ also observed the values of *K*_*f*_ (2.89) and *n*_F_ (1–10) to be more than 1. The obtained results from Freundlich models suggests partial heterogeneous sorption of Zn^2+^ on fungal biomass surface in addition to the homogenous sorption.

The *r*^2^ value of Temkin (*r*^*2*^ = 0.85) and D-R (*r*^*2*^ = 0.92) models confirmed the suitability of these models. Relatively low *r*^2^ obtained in Temkin model indicated that the heat of sorption of Zn^2+^ in a layer remained with the surface coverage of sorbate-sorbent interactions^74^. The calculated values of Temkin constants, namely *B* and *b*_*T*_, were found to be 5.67 *±* 0.15 J mol^− 1^ and 441.57, respectively. Kumar et al.^75^ reported *B* value being less than 219 J mol^− 1^, signifies that the sorption process is primarily physical. Moreover, the positive value of *b*_*T*_ indicates that the enthalpy change of the sorption process is exothermic. These results confirmed the physisorption of Zn^2+^ onto the fungal biomass surface.

D-R model is a measure of biosorption energy^45^. Notably, the *E* value of sorption free energy (0.66 KJ mol^− 1^) obtained from the D-R model was found to be significantly less than 8 KJ mol^− 1^, which indicated that physical sorption played a major role in the removal of Zn^2+^ by *A. terreus* SJP02^45,70^. Shokoohi et al.^68^ also reported similar observations while studying the removal of Cr (VI) and Cd (II) using *A. terreus*.

The fitting of experimental data with all the tested isotherms confirmed the physical and combination of homogeneous and heterogeneous sorption Zn^2+^ on *A. terreus* SJP02 surface. Physical sorption involves attraction forces like van der Waals and electrostatic interactions which are generally considered as relatively weak interactions between the sorbate and sorbent, thereby providing opportunity for the desorption of Zn^2+^ from biosorbent surface^3^. It would further help in safe disposal/ utilization of spent mycosorbent.

#### Kinetic models

**Fig. 8:**
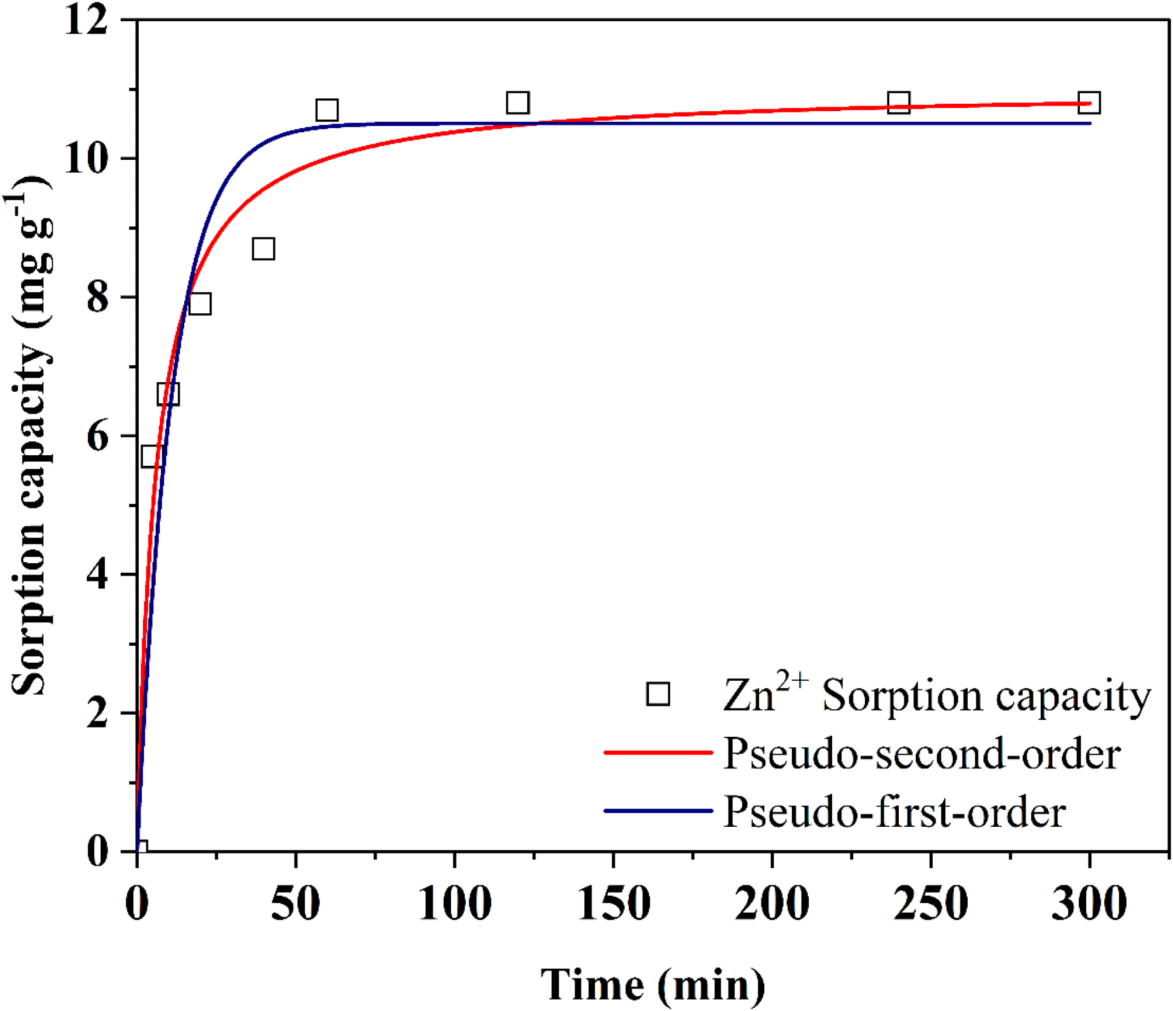
Pseudo 1st order and 2nd second order for the kinetic data of Zn^2+^ removal by *A. terreus* SJP02.

The non-linear form of pseudo-first-order and pseudo-second-order kinetic models were used to fit the experimental results (Fig. 8). The obtained *r*^2^ values with kinetic parameters are shown in Table 3. The pseudo-second-order showed a better fit with *r*^2^ values of 0.97 in comparison to the pseudo-first-order model (0.93). Maximum sorption capacities of 10.27 and 11.01 mg g^− 1^ were obtained from pseudo-first-order and pseudo-second-order kinetic models, respectively, which were found to be quite close to the obtained experimental value of 10.7 *±* 0.2 mg g^− 1^ (biomass dose: 8 g; contact time: 60 min; temperature: 28 ºC; pH: 5.65; initial Zn^2+^concentration: 100 mg L^− 1^). The pseudo-second-order model was found to be suitable with a lower initial concentration of Zn^2+^ and suggests the rate-controlled sorption of Zn^2+^ onto the biomass surface of *A. terreus* SJP02^45^. While studying the bioremediation of Cr^6+^ by *Rhizopus* sp., Espinoza-Sánchez et al.^35^ also reported suitability of pseudo-second-order model for lower initial concentration of Cr^6+^. Conclusively, the sorption of Zn^2+^on *A. terreus* SJP02 surface was rapid which makes it a promising candidate for the industrial scale remediation of Zn^2+^ contaminated wastewater.

#### Comparison with other mycosorbents

In the present study, fungal biomass of *A. terreus* SJP02 exhibited maximum Zn^2+^ sorption of 10.7 *±* 0.02 mg g^− 1^, with an equilibrium time of 60 min. Table 4 represents comparison of maximum Zn^2+^ sorption capacity of *A. terreus* SJP02 with other reported live fungal biomass as mycosorbent for Zn^2+^ removal in aqueous solution. Li et al.^26^ reported higher Zn^2+^ sorption capacity (42.75 mg g^− 1^) using live cells of *Streptomyces ciscaucasicus* with an equilibration time of 24 h. Similarly, Liu et al.^53^ reported Zn^2+^ sorption capacity of 23.70 mg g^− 1^ with equilibrium time of 24 h by the filamentous fungi *Aspergillus niger*. Yan and Viraraghavan^76^ reported a Zn^2+^ sorption capacity of 7.75 mg g^− 1^ in 12 h for *Mucor rouxii*. However, with a much lower Zn^2+^ sorption capacity (2.96 mg g^− 1^), Gunjal et al.^77^ found that *Aspergillus oryzae* could sequester metal ions within 20 min of equilibrium time. A careful comparison with the earlier reports suggested that the fungal biosorbent reported in the present study manifested good sorption capacity within very short time of only 1 h. It implies that the previously reported fungal biomass either had a lower sorption capacity or required longer equilibrium time up to 24 h for the removal of Zn^2+^ from aqueous solution. Present study results suggest that *A. terreus* SJP02 has the potential to be used as a promising biosorbent for Zn^2+^ remediation at industrial scale. Moreover, the present is first study on utilization of fungal mycelia in the “ball form” for the bioremediation purpose. In the ball form, mycelia are easier to handle and offer more opportunities for large-scale industrial applications. After desorption of Zn^2+^, the spent biomass can be incinerated and used as fertilizer and/ or biochar to improve the soil quality^78–80^.

**Table 4:** Comparison of maximum Zn^2+^ ions adsorption capacity of *aspergillus terreus* SJP02 with other reported live fungal biosorbents.

Fungal species used as biosorbent	Q_max_ (mg g^-1^)	Parameters	References
*Mucor rouxii*	7.75	*C*_*0*_: 10 mg L^− 1^, *m*: 0.2 g, pH: 5.0, *t*: 12 h	Yan and Viraraghavan^76^
*Aspergillus niger*	23.70	*C*_*0*_: 150 mg L^− 1^, *m*: 0.2 g, pH: 6.0, *t*: 24 h	Liu et al.^53^
*Streptomyces circauscasicus*	42.75	*C*_*0*_: 150 mg L^− 1^, *m*: 1.5 g, pH: 5.0, *t*: 24 h	Li et al.^26^
*Aspergillus oryzae*	2.96	*C*_*0*_: 150 mg L^− 1^, *m*: 0.5 g, pH: 5.0, *t*: 20 min	Gunjal et al.^77^
*Aspergillus terreus* SJP02	10.7 *±* 0.2	*C*_*0*_: 100 mg L^-1^, *m*: 0.184 g, pH: 5.65, *t*: 1 h	Present study

*Q*_*max*_ maximum sorption capacity, *C*_*0*_ metal concentration, *m* mass in dry weight, *t* time.

Pilot scale column studies using optimized batch study parameters are under investigation to treat the Zn^2+^ loaded industrial effluents.

## Conclusions

The present study demonstrates utilization of live fungal biomass of *A. terreus* SJP02 for the efficient removal of Zn^2+^ from aqueous solution. A series of experiments were conducted to study the effect of various process parameters viz. biosorbent dose, contact time, temperature, agitation rate, pH, and initial concentration of Zn^2+^ on the fungal sorption capacity. Maximum Zn^2+^ sorption was observed at 28 ºC which is the well-known optimum temperature required for the fungal growth. Notably, among tested pH conditions, the fungal strain demonstrated maximum growth at pH 5.65, which is the natural pH of Zn^2+^solution (100 mg L^− 1^). Utilization of fungal biomass in the ball form can certainly reduce their immobilization costs, which is a significant barrier in scaling up at industrial level. Desorption studies conducted to recover Zn^2+^from the spent mycosorbent showed a recovery rate of 71.46% within 120 min. The acid digestion studies suggested that the cell wall adsorption of Zn^2+^ was the primarily mechanism behind removal of Zn^2+^ from aqueous solution, which was further confirmed by the FE-SEM, EDX and FTIR analysis. Equilibrium isotherms and kinetic models also suggested involvement of physical adsorption processes in Zn^2+^ removal. It provides opportunity for the efficient metal recovery for reuse and reutilization of spent mycosorbent, which would be economically advantageous for the industrial scale applications. The comparison of *A. terreus* SJP02 biomass with the other reported mycosorbents noticeably signifies it’s potential and makes it a promising candidate for the industrial scale remediation of Zn^2+^ contaminated wastewater. Pilot scale column studies using optimized batch study parameters are under investigation to treat the Zn^2+^ loaded industrial effluents.

## Electronic supplementary material

Below is the link to the electronic supplementary material.

Supplementary Material 1

## Data Availability

The authors declare that the data supporting the study are available in the article. If any raw data files in other formats are required, they can be obtained from the corresponding author upon request. The ITS1-5.8 S-ITS2 gene complex sequence is submitted in the NCBI GenBank database (Accession number OR726084). The fungal isolate has also been deposited in the Microbial Type Culture Collection (MTCC) at the Institute of Microbial Technology (IMTech), Chandigarh, India, and is available in the public domain with the MTCC number 13417.
