# Supplementary material for: Unveiling the potential of Aspergillus terreus SJP02 for zinc remediation and its driving mechanism
Source: Sci Rep. 2025 Jan 27;15:3376. doi: 10.1038/s41598-025-87749-3 (PMC11772822; doi:10.1038/s41598-025-87749-3)
Supplement: Supplementary file 1 — Supplementary Material 1 [file 41598_2025_87749_MOESM1_ESM.docx]

**Supplementary Materials**

**Supplementary Table S1: Concentration of heavy metals in the rhizospheric soils of Mandideep industrial area, Bhopal, India.**

| Heavy metal | Value (mg kg^-1^) | WHO permissible limit (mg/kg) in contaminated soil |
| --- | --- | --- |
| Co | 96 + 0.8 | 40 |
| Ni | 161 + 1.7 | 35 |
| Pb | 54 + 2.7 | 85 |
| Cu | 1073 + 0.7 | 36 |
| Zn | 1015.3 + 1.4 | 50 |
| Cr | 137.5 + 0.5 | 100 |

**Supplementary Table S2: Screening of fungal isolates for Zn^2+^ removal.**

| Fungal isolate | Percent Zn^2+^ removal  after 24 h |
| --- | --- |
| SJP01 | 6.56 + 1.41 |
| SJP02 | 25.86 + 4.21 |
| SJP03 | 23.04 + 3.42 |
| SJP04 | 10.10 + 0.59 |
| SJP05 | 20.53 + 2.73 |
| SJP06 | 15.04 + 4.19 |
| SJP07 | 12.57 + 3.14 |
| SJP08 | 6.04 + 0.96 |
| SJP09 | 10.54 + 4.55 |
| SJP10 | 19.76 + 1.13 |
| SJP11 | 3.28 + 2.01 |
| SJP12 | 7.45 + 0.44 |
| SJP13 | 7.29 + 1.06 |
| SJP14 | 3.80 + 1.56 |
| SJP15 | 16.46 + 2.38 |

**Supplementary Table S3:** Sorption capacity (q) of live vs dead biomass of *A. terreus* SJP02 for Zn^2+^ removal from aqueous solution.

| Biomass | Sorption capacity  (mg g^-1^) |
| --- | --- |
| Live | 10.7 + 0.2 |
| Dead | 9.5 + 0.3 |

**Supplementary Table S4:** EDX analysis of untreated (control), Zn treated and Zn desorbed biomass of *A. terreus* SJP02.

| Map Sum Spectrum | Untreated biomass  (atomic %) | Zn treated biomass (atomic %) | Zn desorbed biomass (atomic %) |
| --- | --- | --- | --- |
| C | 79.43 | 80.65 | 67.06 |
| O | 20.55 | 18.04 | 32.94 |
| Zn | 0.02 | 1.31 | 0.00 |
| Total | 100.00 | 100.00 | 100.0 |


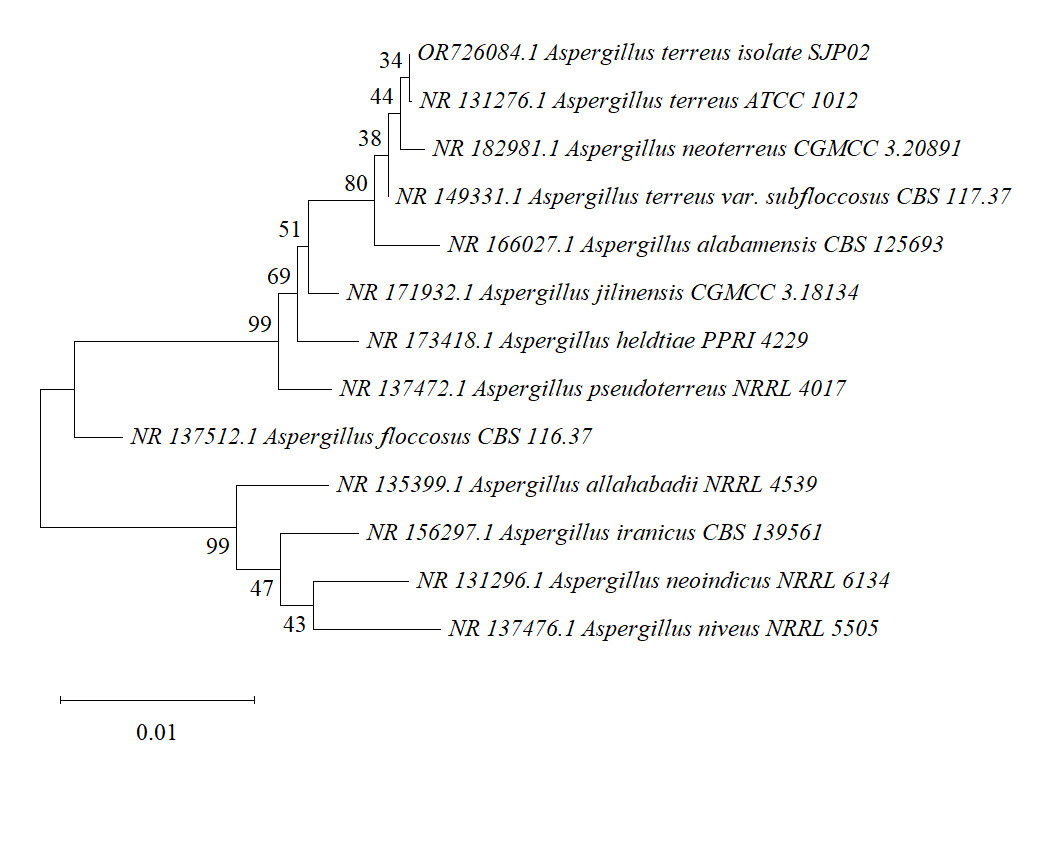


**Supplementary Figure S1:** The phylogenetic position of *Aspergillus terreus* isolate SJP02, along with closely related species of the genus *Aspergillus*, is depicted in the tree. The bootstrap support values, indicating the percentage of replicate trees where the associated taxa clustered together, are displayed adjacent to the branches. The scale bar (0.01) represents the genetic distance.
